# Real-Time Wing Deformation Monitoring via Distributed Fiber Bragg Grating and Adaptive Federated Filtering

**DOI:** 10.3390/s25144343

**Published:** 2025-07-11

**Authors:** Zhen Ma, Xiyuan Chen, Cundeng Wang, Bingbo Cui

**Affiliations:** 1 School of Agricultural Engineering, Jiangsu University, Zhenjiang 212013, China; 1000005769@ujs.edu.cn; 2 School of Instrument Science and Engineering, Southeast University, Nanjing 210096, China; chxiyuan@seu.edu.cn (X.C.); 230239006@seu.edu.cn (C.W.)

**Keywords:** FBG, wing deformation, distributed transfer alignment, filtering

## Abstract

To address the issues of decreased accuracy and poor stability in distributed transfer alignment caused by factors such as wing deflection and deformation in complex flight environments, this paper proposes a wing-distributed transfer alignment method based on Fiber Bragg Grating (FBG). This paper establishes a flexural deformation model based on FBGs, establishes a coupling angle model and a dynamic lever arm model, derives the motion parameter relationship model between the main and the sub-nodes, establishes the corresponding transfer alignment filter, and proposes a federated adaptive filter based on allocation coefficients and an updated federated adaptive filter. The results show that the federated adaptive filtering algorithm based on allocation coefficients improved the pitch angle accuracy of the Inertial Measurement Unit (IMU) by 66.38% and the position estimation accuracy by 75.67%, compared to traditional algorithms. The arm estimation accuracy was also improved in the east and sky directions. Compared with traditional algorithms, the updated federated adaptive filtering algorithm improved the pitch angle accuracy of the sub IMU by 76.72%, the position estimation accuracy by 63.51%, and the lever arm estimation accuracy.

## 1. Introduction

The distributed pose measurement system can provide distributed high-precision motion information for multiple payloads in high-resolution aerial ground observation systems. However, the observation platform is affected by loads and turbulence during flight, which causes wing deflection and vibration, thereby reducing the accuracy of alignment between the primary and secondary nodes [1,2]. Improving the accuracy of distributed transfer alignment has become the key to high-resolution imaging of aerial to ground observation systems. The traditional transfer alignment error model that does not consider the dynamic deformation of the primary and secondary nodes, as well as the distributed Kalman filtering method that does not consider the time-varying characteristics of noise and information fusion between primary and secondary nodes, are difficult to provide high-precision information when used for distributed transfer alignment [3,4,5]. The traditional transfer alignment error model treats the wing as a rigid body and ignores the dynamic deformation between the primary and secondary nodes, which hinders the achievement to achieve high-precision measurement [6,7].

References [8,9] used second-order Markov processes to simulate dynamic bending deformation angles and added relevant bending deformation angles and angular velocities as state variables. However, the models treated the dynamic lever arm as constant, which does not conform to actual deformation. Reference [10] established a model between deformation angle and dynamic lever arm and conducted a detailed analysis of the velocity relationship between the primary and secondary nodes, including the coupling relationship. In airborne-distributed transfer alignment, wing deformation can increase the estimation error of the lever arm, and attitude and angular velocity matching methods can avoid it [11]. The coupling relationship between the angular velocities of the main and sub-nodes, specifically the coupling angle caused by the wing deflection deformation and the direction of the angular velocity of the sub-nodes, also needs to be considered.

When establishing the alignment error model, the deflection deformation angle, the coupling angle, and the dynamic arm error of the main and secondary nodes of the wing are considered. In practical engineering applications, although more accurate models are used, the interference of complex flight environments can lead to a decrease in the accuracy of Kalman filtering, and even filter divergence [12,13,14,15]. Adaptive filtering methods have become a research focus. Reference [16] proposes an adaptive Kalman filter based on the maximum likelihood estimation criterion of new information, which can estimate the system process noise matrix Q and measurement noise matrix R in real time, and can better estimate the misalignment angle. However, Q and R must be estimated continuously, and the large computational complexity can lead to a decrease in real-time performance. Therefore, considering the accuracy and time of transfer alignment, a method of continuously estimating only R is adopted, but the state estimation error can affect the stability of this filtering method. The residual based chi square test [17] can effectively solve this problem. Therefore, this paper introduces the Kalman filtering method based on the R update and the covariance matrix P update based on the chi square test for single-point transfer alignment. The single adaptive Kalman filter has poor anti-interference performance when used for distributed transfer alignment, and different sub-filters can be fused to achieve better results [18,19]. In the multi-node information fusion method, federated filtering is widely used due to its good fault tolerance. It improves the federated filter by real-time determination of information weight coefficients [20,21].

The above research shows that there are three methods for compensating lever arm errors: the first method directly calculates acceleration and velocity error compensation using lever arm values, but this method requires accurate lever arm length, which has certain limitations. The second method analyzes the frequency spectrum of the lever arm that needs to be compensated and uses low-pass filtering to filter out low-frequency lever arm disturbances and eliminate the influence of the lever arm. This method is simple, but digital filters have delay phenomena, which limits accuracy. The third method adds the lever arm that needs to be compensated as a state variable to the system model, which can estimate the length change of the lever arm in real time. After adding installation errors and scale factors to the state matrix, the estimation accuracy can be further improved. This paper combines the background of distributed transfer alignment and complex flight environments, aiming to improve the accuracy and stability of transfer alignment, shorten the time and stability of transfer alignment, and propose a new federated adaptive filtering method for distributed transfer alignment by combining FBG sensing technology. The sub-filters adopt Kalman filtering based on R and P adaptive updates, combined with a dimensional transfer alignment model for single-point transfer alignment. The main filter uses the principle of norm-based error covariance matrix to fuse information from multiple sub-filters.

## 2. Materials and Methods

**Figure 1:**
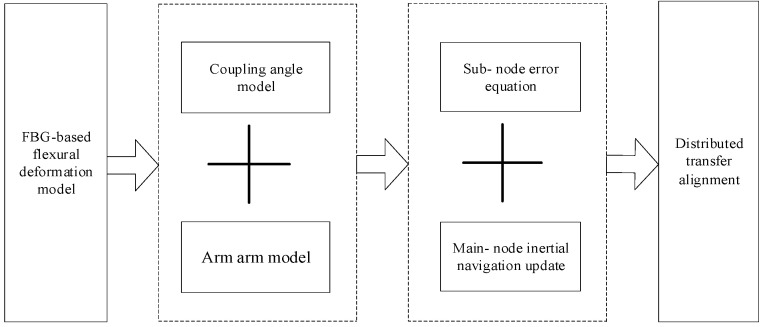
Route of airborne distributed transfer alignment technology.

The working principle of FBG sensors is actually to utilize changes in the physical environment surrounding the FBG sensor, such as stress, strain, temperature, etc., to form grating periods or changes in the refractive index of the fiber core [22,23]. By measuring the shift of the center wavelength of FBG, the variation law of stress-strain can be obtained [24,25]. Due to the low accuracy of sub IMU and the complex flexible deformation between sub-nodes, it was necessary to study the carrier model. Through FBG measurements of flexible wing deformation and transfer alignment, main and sub-information fusion was achieved to achieve accurate measurements of the distributed array antenna flexible multi-baseline. This section uses FBG to assist in establishing an accurate transfer alignment model. The technology roadmap is shown in Figure 1.

### 2.1. FBG Layout Packaging Design and Implementation

#### 2.1.1. FBG Layout Design

In order to effectively analyze the distributed deformation measurement method of wings in airborne environments, an indoor distributed measurement system platform needs to be built before real flight testing [26]. Sensors are typically designed based on optimization criteria, such as reliability, observability, and interpolation fitting. The layout design of sensors is not only related to the shape of the object being measured, but also to the working conditions and main force forms, which can affect the layout scheme of the sensor. Considering that the application background of this paper is wing deformation measurements, a single FBG cannot obtain the overall deformation information of the wing. It is necessary to effectively use multiplexing technology to determine the layout network. At the same time, the arrangement direction of the sensor array should be aligned with the bending direction of the wing under stress, and the installation position of the sensor array should be arranged according to the thickness of the wing. The sensor arrays should be arranged in a parallel and non-equidistant manner. The FBG array designed in this paper senses the deformation of the wing and calculates the spatial position coordinates of each node after deformation. Therefore, the sensor layout also needs to consider the installation position of the distributed IMU.

**Figure 2:**
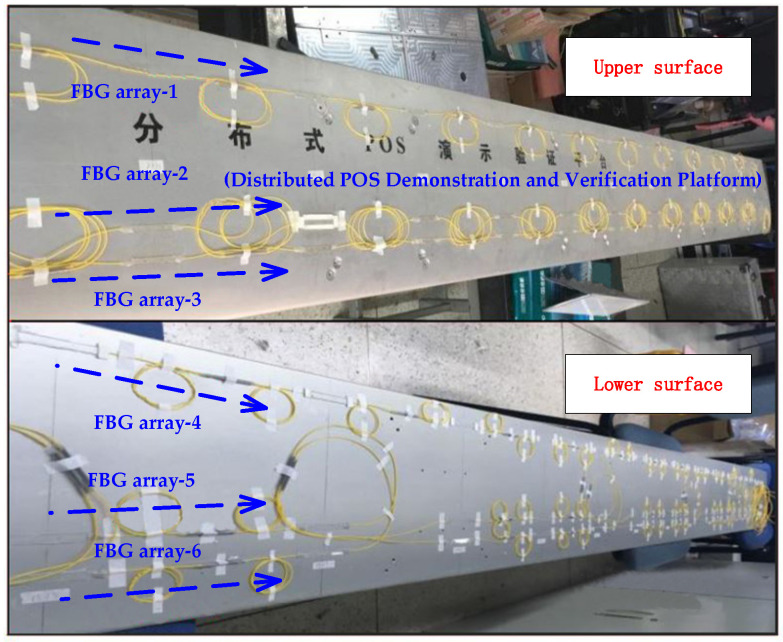
Layout scheme of an airborne FBG array.

In order to ensure the accuracy of the airborne FBG measurement system, it is necessary to design the quantity and position of FBGs laid on the wing surface reasonably. According to the mechanical properties of the wing, it is known that there is a significant change in stress at the root of the wing during bending deformation, so it is necessary to increase the density when arranging FBG nodes at the root of the wing. Combining FBG multiplexing technology, this paper adopts a combination of WDM technology and SDM technology to build an airborne FBG layout network [27], as shown in Figure 2. Three FBG arrays are symmetrically arranged on the upper and lower surfaces of the wings, and a total of 12 FBG arrays are laid on two pairs of wings, with each array parallel to each other.

The number and spacing of grating nodes in each FBG array layout designed in this article are related to the strain sensitivity coefficient $K_{\varepsilon }$ and wavelength buffer zone $\lambda_{k}$ of the sensor. The wavelength buffer zone refers to the wavelength difference between two adjacent gratings on an FBG array; that is, the interval between the maximum wavelength of the former and the minimum wavelength of the adjacent latter. A reasonable design of the wavelength buffer zone $\lambda_{k}$ is conducive to improving measurement accuracy while ensuring that the center wavelength values of any two measurement points do not overlap. The size of the buffer directly affects the number of raster nodes on an array within a limited range.

Assuming there are *M* grating nodes on an FBG array and the strain sensitivity coefficient and wavelength buffer of each sensor are the same, the total measurement interval length $\lambda_{k}$ of the M grating nodes are as follows:

(1) $$\lambda_{M}=\sum_{i=1}^{M}\left(K_{\varepsilon }\varepsilon_{i}+\lambda_{k}\right)=\sum_{i=1}^{M}K_{\varepsilon }\varepsilon_{i}+M\lambda_{k}$$

According to the measurement requirements, the total measurement interval length $\lambda_{M}$ should be within the maximum range $\lambda_{\max}$ as follows:

(2) $$\lambda_{M}\leq \lambda_{\max}$$

The following formula can be obtained by combining Formulas (1) and (2):

(3) $$\sum_{i=1}^{M}K_{\varepsilon }\varepsilon_{i}+M\lambda_{k}\leq \lambda_{\max}$$

(4) $$M\leq \frac{\left(\lambda_{\max}-\sum_{i=1}^{N}K_{\varepsilon }\varepsilon_{i}\right)}{\lambda_{k}}$$

The range of values for the number of grating nodes on a single FBG array can be determined by Formula (4). In practical engineering applications, sufficient center wavelength spacing should be left within the wavelength range of the experimental demodulator to ensure that the center wavelengths of adjacent nodes do not overlap. The demodulator selected in this article has a wide measurement range and a large center wavelength interval can be set. Therefore, the difference between adjacent grating nodes is set to 4 nm, and so on. Each FBG array has a total of 14 nodes, and 12 arrays have a total of 168 nodes. Array-2 and array-5 are arranged at the position with the largest wing thickness, with a distance of 106 mm from the leading edge of the wing, and a distance of 135 mm between array-1 and array-3, as shown in Figure 2.

#### 2.1.2. FBG Packaging Design

**Figure 3:**
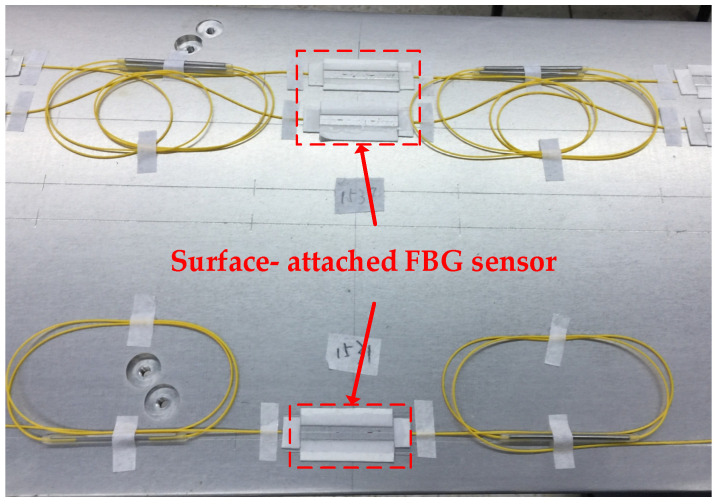
Packaging method of FBG sensor.

Tube-encapsulated FBGs need to be embedded in the tested structure, which can damage the surface structure of the aircraft wing [28]. Embedded encapsulated FBGs have a large volume and limit the number of FBG nodes arranged in a limited space. Surface-mounted FBGs are suitable for surface strain sensing but have higher requirements for the bonding process of FBGs. Based on the above analysis, combined with the advantages of simple production, simple structure, and suitability for carrier surface strain monitoring of surface-mounted FBGs, surface-mounted FBGs were selected as the packaging form for the sensors used in this article in the indoor platform design. The physical graphics are shown in Figure 3. Before packaging, the exposed FBG and adhesive positions should be cleaned with alcohol. Then, the bonding area should be sanded with sandpaper to increase the adhesion of the bond. The bonding position should be marked with a steel ruler, and the surrounding coils fixed with tape. The adhesive should be mixed evenly, and the optical fiber should be kept straight when pasting.

### 2.2. FBG Assisted Transfer Alignment

#### 2.2.1. The Bending Deformation Angle Model Based on FBGs

**Figure 4:**
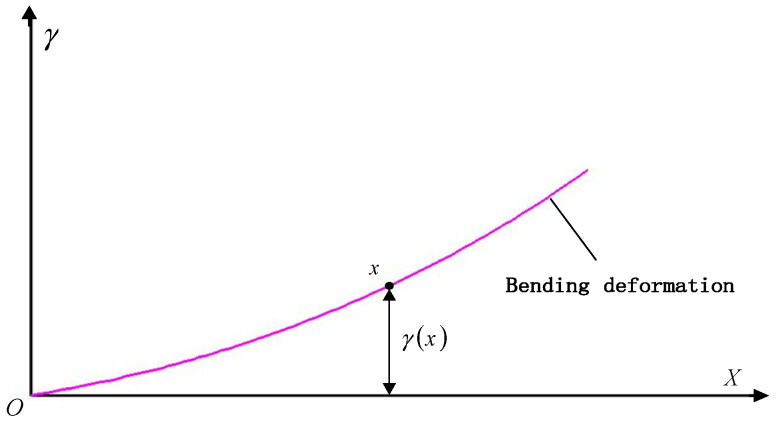
Schematic diagram of bending deformation.

As shown in Figure 4, assuming the wing extension direction as the *X*-axis, the time-varying deflection value γ and the time-varying angle $\theta^{b}$ at any point on the cantilever beam model can be expressed as follows [20]:

(5) $$\left\{\begin{array}{l} \gamma \left(x,t\right)=\frac{Fx^{2}}{6EI}\left(3l-x\right)\\ \theta^{b}\left(x,t\right)=\frac{Fx}{2EI}\left(2l-x\right) \end{array}\right.$$

By combining the load *F* and strain ε relationship, the relationship between the strain $\varepsilon_{x}$ at point x and the time-varying deflection γ and deformation angle $\theta^{b}$ can be derived.

(6) $$\left\{\begin{array}{l} \gamma \left(x,t\right)=\frac{x^{2}}{3b}\cdot \frac{\left(3l-x\right)}{\left(l-x\right)}\varepsilon_{x}\\ \theta^{b}\left(x,t\right)=\frac{x}{b}\cdot \frac{\left(2l-x\right)}{\left(l-x\right)}\varepsilon_{x} \end{array}\right.$$

The relationship between wavelength variation ${∆\lambda }_{B(x)}$ and strain $\varepsilon_{x}$ at any point x on the wing can be expressed as follows:

(7) $$\varepsilon_{x}=\frac{1}{\left(1-P_{e}\right)}\cdot \frac{∆\lambda_{B(x)}}{\lambda_{B(x)}}$$

The relationship between the time-varying deflection γ and the time-varying deformation angle $\theta^{b}$ expressed by the wavelength change ${∆\lambda }_{B(x)}$ at any point x on the wing is as follows:

(8) $$\left\{\begin{array}{l} \gamma \left(x,t\right)=\frac{1}{\left(1-P_{e}\right)}\cdot \frac{x^{2}}{3b}\cdot \frac{\left(3l-x\right)}{\left(l-x\right)}\cdot \frac{∆\lambda_{B(x)}}{\lambda_{B(x)}}\\ \theta^{b}\left(x,t\right)=\frac{1}{\left(1-P_{e}\right)}\cdot \frac{x}{b}\cdot \frac{\left(2l-x\right)}{\left(l-x\right)}\cdot \frac{∆\lambda_{B(x)}}{\lambda_{B(x)}} \end{array}\right.$$

where, the photoelastic coefficient $P_{e}$, wing thickness b, wing length l, and center wavelength ${∆\lambda }_{B(x)}$ at point x are all constants. Therefore, the time-varying deflection γ and time-varying angle $\theta^{b}$ at any node on the wing can be directly derived from the wavelength change ${∆\lambda }_{B(x)}$.

#### 2.2.2. Coordinate System Description

Due to the unique operating environment of the aircraft, the relative positions of the phase centers of various observation loads cannot be determined. To obtain high-precision motion parameters of the phase centers of each sub-observation load in the aviation to ground observation system, it is necessary to study high-precision transfer alignment methods between the main and sub-systems in the distributed measurement systems [29]. The motion relationship between objects is relative, and the study of carrier motion involves the definition of coordinate systems. The following are commonly used coordinate systems in inertial navigation research and their expressions in this article.

(1) Geocentric Inertial Coordinate System ($O_{i}X_{i}Y_{i}Z_{i}$, i-System)

**Figure 5:**
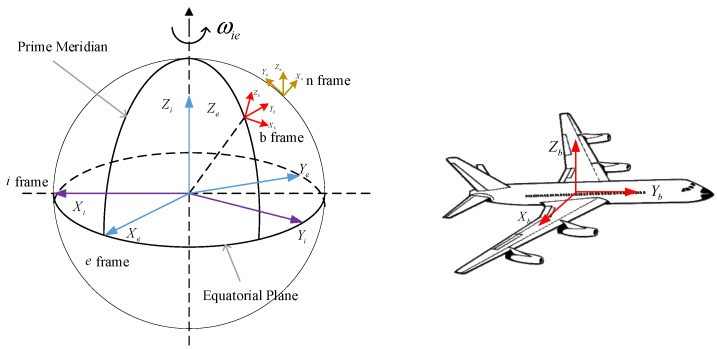
Schematic diagram of the carrier coordinate system.

A spatial coordinate system is established with the Earth’s center of mass as the origin O, where the intersection line between the *X*-axis and the first meridian plane coincides, and the east direction is positive; the *Z*-axis coincides with the Earth’s rotation axis and points towards the North Pole as positive along the Earth’s axis; and the *Y*-axis is perpendicular to the XZ plane and follows the right-hand coordinate system principle, as shown in Figure 5. Due to the use of different epoch times, there can be various geocentric inertial coordinate systems. Currently, the internationally recognized geocentric inertial coordinate system is the J2000 epoch coordinate system, which is based on the vernal equinox in the year 2000.

(2) Earth-Centered Earth-Fixed Coordinate System ($O_{e}X_{e}Y_{e}Z_{e}$, e system)

The coordinate origin O of the earth-centered, earth-fixed coordinate system is located at the center of the earth, and the *X*-axis is located in the equatorial plane and points to the geographic longitude zero point; the *Z*-axis points towards the North Pole of the Earth; and the *Y*-axis is perpendicular to the XZ plane and follows the right-hand coordinate system principle. The geocentric coordinate system is the Cartesian Cartesian Cartesian coordinate system, which is stationary relative to the Earth in space, accompanied by the Earth’s rotation and revolution.

(3) Navigation coordinate system ($O_{n}X_{n}Y_{n}Z_{n}$, *n* system)

The navigation coordinate system, as an important coordinate system for solving navigation parameters, has its origin as the centroid of the carrier. In the strapdown inertial navigation system, the output of the inertial device is generally the measured value in the Body Coordinate System, but the navigation parameters are not solved in the Body Coordinate System. Instead, the measured value of the inertial device is decomposed into a selected navigation coordinate system for navigation calculation, which is the navigation coordinate system. The attitude determined by the Body Coordinate System relative to the navigation coordinate system can be represented by pitch angle, roll angle, and yaw angle.

(4) Body Coordinate System ($O_{b}X_{b}Y_{b}Z_{b}$, b system)

The origin of the body coordinate system coincides with the center of gravity of the carrier, with the *X*-axis pointing to the right along the carrier, the *Y*-axis pointing forward along the carrier, and the *Z*-axis pointing upward along the carrier. The three axes form a right-handed Cartesian coordinate system, as shown in Figure 5. In this article, the coordinate system of the main system carrier of the distributed inertial measurement system is represented by m; and the coordinate system of the subsystem carrier is represented by s.

#### 2.2.3. Coupling Error Angle Model

Assuming that the sub-nodes have no errors, their angular velocity can be expressed as follows:

(9) $$\left\{\begin{array}{c} \omega =\omega_{g}+\omega_{t}\\ \omega_{t}=\omega_{t}^{b}+\omega_{t}^{z} \end{array}\right.$$

where ω represents the error free angular velocity of the sub-nodes, $\omega_{g}$ represents the angular velocity generated by rigid body motion, $\omega_{t}$ represents the angular velocity caused by elastic deformation, $\omega_{t}^{b}$ represents the angular velocity caused by bending deformation of the middle wing, and $\omega_{t}^{z}$ represents the angular velocity component caused by middle vibration. The angular velocity caused by wing vibration is treated as white noise.

(10) $$\omega_{t}^{b}=\omega_{\theta }={\dot{\theta }}^{b}{\left(x,t\right)}_{t}$$

The formula for the ideal error angle between the main node and the child node is shown as follows:

(11) $$\mu_{\theta }=\rho_{0}+\theta^{b}\left(x,t\right)$$

where $\rho_{0}$ represents the initial installation error angle between the main node and the sub-node. In the ideal state of the Earth-centered inertial coordinate, the angular velocity formula of the sub-nodes is shown as follows:

(12) $$\omega_{is}^{s}=C_{m}^{s}(\mu_{\theta })\omega_{im}^{m}$$

where $C_{m}^{s}(\mu_{\theta })$ represents the direction cosine matrix from the main node to the sub-node in the ideal state, and $\omega_{im}^{m}$ represents the angular velocity of the main node relative to the geocentric coordinate in the main node coordinate system.

**Figure 6:**
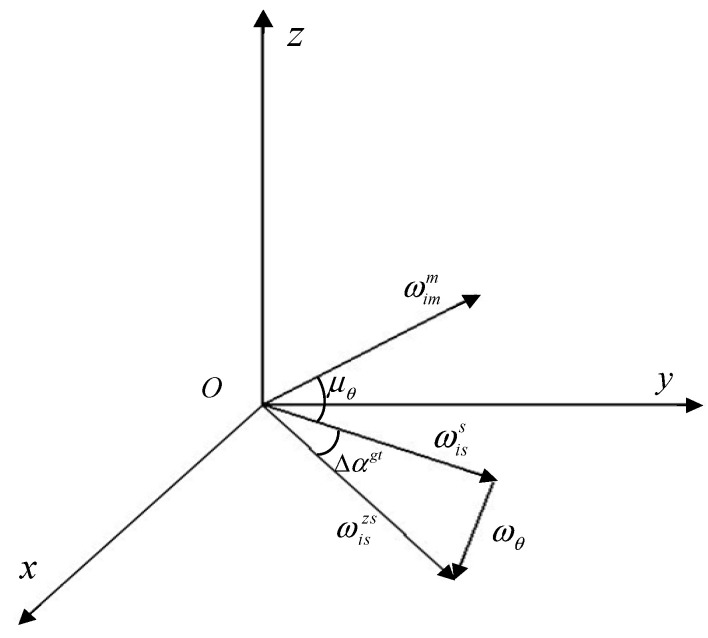
Angular velocity relationship of the main and sub-nodes.

In the real state, as shown in Figure 6, due to the non-collinearity between the ideal angular velocity $\omega_{is}^{s}$ of the sub-node and the angular velocity $\omega_{\theta }$ caused by bending deformation, there will be a coupling error angle $∆\alpha^{gt}$ caused by the combination of rigid body motion and bending deformation between the main and sub-nodes. The angular velocity of the sub-node in the real state should be expressed as follows:

(13) $$\omega_{is}^{zs}=\omega_{is}^{s}+\omega_{\theta }$$

where $\omega_{is}^{s}={\left[\begin{array}{ccc} \omega_{isx}^{s} & \omega_{isy}^{s} & \omega_{isz}^{s} \end{array}\right]}^{T}$, $\omega_{\theta }={\left[\begin{array}{ccc} \omega_{\theta x} & \omega_{\theta y} & \omega_{\theta z} \end{array}\right]}^{T}$, the subscripts x, y, and z respectively, represent the direction of the ENU (East-North-Up Coordinate System), which can be obtained as follows:

(14) $$\omega_{is}^{s}={\left[\begin{array}{ccc} \omega_{isx}^{zs}-\omega_{\theta x} & \omega_{isy}^{zs}-\omega_{\theta y} & \omega_{isz}^{zs}-\omega_{\theta z} \end{array}\right]}^{T}$$

The coupling error angles in three directions can be expressed as follows:

(15) $$∆\alpha^{gt}={\left[\begin{array}{ccc} ∆\alpha_{x}^{gt} & ∆\alpha_{y}^{gt} & ∆\alpha_{z}^{gt} \end{array}\right]}^{T}$$

Taking the x-axis as an example, the ideal angular velocity of the sub-node is represented by $\omega_{isx}^{s}$, the actual angular velocity is represented by $\omega_{isx}^{zs}$, the coupling error angle is represented by $∆\alpha^{gt}$, and the deformation angular velocity is represented by $\omega_{\theta x}$. The coupling error angle in the x-axis can be expressed as follows:

(16) $$\begin{array}{ll} ∆\alpha_{x}^{gt} & =arctan\frac{\omega_{isz}^{zs}}{\omega_{isy}^{zs}}-arctan\frac{\omega_{isz}^{s}}{\omega_{isy}^{s}}\\ & =arctan\frac{\omega_{isz}^{zs}}{\omega_{isy}^{zs}}-arctan\frac{\omega_{isz}^{zs}-\omega_{\theta z}}{\omega_{isy}^{zs}-\omega_{\theta y}} \end{array}$$

According to the small coupling error angle, the formula can be expanded by the Taylor series, and the following can be obtained as follows:

(17) $$∆\alpha_{x}^{gt}\approx \frac{\omega_{isz}^{zs}}{\omega_{isy}^{zs}}-\frac{\omega_{isz}^{zs}-\omega_{\theta z}}{\omega_{isy}^{zs}-\omega_{\theta y}}$$

Similarly, the coupling error angle formula for the *y*-axis and *z*-axis directions can be obtained as follows:

(18) $$\left\{\begin{array}{l} ∆\alpha_{y}^{gt}\approx \frac{\omega_{isx}^{zs}}{\omega_{isz}^{zs}}-\frac{\omega_{isx}^{zs}-\omega_{\theta x}}{\omega_{isz}^{zs}-\omega_{\theta z}}\\ ∆\alpha_{z}^{gt}\approx \frac{\omega_{isy}^{zs}}{\omega_{isx}^{zs}}-\frac{\omega_{isy}^{zs}-\omega_{\theta y}}{\omega_{isx}^{zs}-\omega_{\theta x}} \end{array}\right.$$

Considering that the deformation angular velocity $\omega_{\theta }$ is much smaller than the actual angular velocity $\omega_{is}^{zs}$ of the sub-node, Formula (15) can be simplified as follows:

(19) $$∆\alpha^{gt}=M\omega_{\theta }$$

where the coefficient matrix be expressed as this, $M=\left[\begin{array}{ccc} 0 & 0 & \frac{1}{\omega_{isy}^{zs}}\\ \frac{1}{\omega_{isz}^{zs}} & 0 & 0\\ 0 & \frac{1}{\omega_{isx}^{zs}} & 0 \end{array}\right]$.

#### 2.2.4. Dynamic Lever Arm Model

In the process of information sharing, there are significant errors in wing deformation and the relative positions of the main and sub-nodes due to changes in the lever arm. Therefore, effective estimation and compensation of the lever arm are needed, and a mathematical model between the deformation angle and the dynamic lever arm has been established.

**Figure 7:**
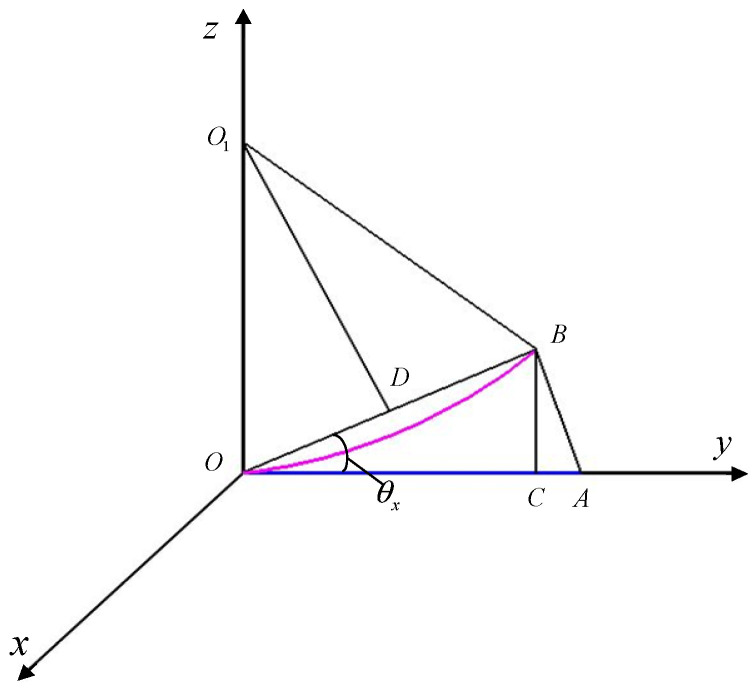
Dynamic lever arm changing along the *y*-axis direction.

Taking $\theta_{x}$ as an example, as shown in Figure 7, ∠*AOB* represents $\theta_{x}$. Assuming that the initial lever arm is $r0={\left[x_{0}\;y_{0}\;z_{0}\right]}^{T}$, the lever arm is a cantilever beam. Section OA represents the initial length of the lever arm; arc OB represents the length of the deformed lever arm; OC and CB, respectively, represent the projection components of the bending deformed lever arm on the y axis and z axis; point $O_{1}$ is the center of the circle corresponding to the arc OB; and triangle ${∆OO}_{1}B$ is an isosceles triangle. Due to the cantilever beam model of the pole arm, it will not stretch or compress. According to the principle of triangular geometry, it can be concluded as follows:

(20) $$\left\{\begin{array}{l} OB=2OD=2OO_{1}\sin\theta_{x}=y_{0}\sin\theta_{x}/\theta_{x}\\ OC=OB\;\cos\theta_{x}=y_{0}\sin\theta_{x}\cos\theta_{x}/\theta_{x}\\ CB=OB\;\sin\theta_{x}=y_{0}\sin^{2}\theta_{x}/\theta_{x} \end{array}\right.$$

From the above equation, it can be seen that the change in lever arm caused by $\theta_{x}$ and the length of lever arm changed by $\theta_{y}$ and $\theta_{z}$ can be obtained.

Therefore, the length of the lever arm can be expressed as follows:

(21) $$r=\left[\begin{array}{l} x_{0}+z_{0}\theta_{y}-2x_{0}\theta_{z}^{2}/3\\ y_{0}+x_{0}\theta_{z}-2y_{0}\theta_{x}^{2}/3\\ z_{0}+y_{0}\theta_{x}-2z_{0}\theta_{y}^{2}/3 \end{array}\right]$$

The Equation (17) ignores the second-order small term and can be updated as follows:

(22) $$r=\left[\begin{array}{l} x_{0}+z_{0}\theta_{y}\\ y_{0}+x_{0}\theta_{z}\\ z_{0}+y_{0}\theta_{x} \end{array}\right]=r_{0}+R_{0}\theta$$

where $R_{0}=\left[\begin{array}{ccc} 0 & z_{0} & 0\\ 0 & 0 & x_{0}\\ y_{0} & 0 & 0 \end{array}\right]$ Considering the influence of the coupling error angle, the dynamic lever arm variation δr can be expressed as follows:

(23) $$\left\{\begin{array}{l} r_{new}=r_{0}+\delta \;r\\ \delta r=R_{0}\left(\theta +∆\alpha^{gt}\right) \end{array}\right.$$

The updated dynamic lever arm has a variation of $\delta r=R_{0}(\theta +\Delta \alpha^{gt})$, which is used for the analysis of the motion parameters of the main and sub-nodes in the next section.

#### 2.2.5. Transfer Alignment Model

**Figure 8:**
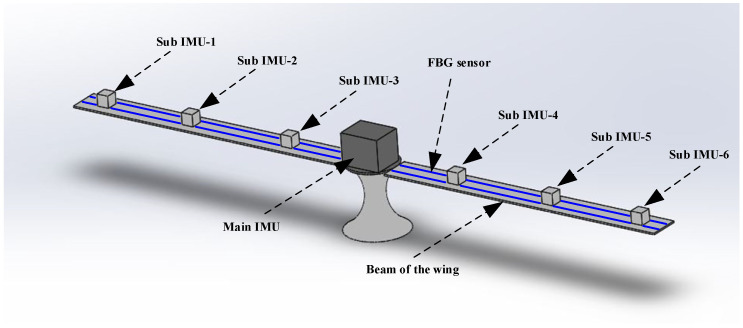
Schematic diagram of the IMU layout on the aircraft wing.

As shown in Figure 8, the main IMU refers to the IMU of the main node in the airborne environment, which has higher accuracy and is usually used as the reference for the system. The sub IMU refers to other nodes in the airborne environment, usually installed in other parts of the aircraft for measurements. To address the issue of inaccurate information transmission between the main and sub-nodes, it is necessary to establish a deflection deformation model based on FBG and, on this basis, derive the coupling angle model and lever arm model and then establish the transfer alignment model.

(1) State equation

Based on previous research, an error state vector is constructed by selecting system state variables such as attitude error, velocity error, position error, gyroscope drift error, and accelerometer bias error. A dynamic equation for the evolution of the system error state vector is established over time by combining the attitude error equation, the position error equation, and the inertial device error model. This section establishes the full-order state equation of the airborne strapdown inertial navigation system based on the error model, as shown below. The Equation of the state can be expressed as follows:

(24) $$\dot{X}=FX+G\varpi$$

where X represents the state vector, F represents the State-transition matrix, and G represents the noise partition coefficient.

(25) $$X=[\Phi \;\delta V\;\varepsilon^{s}\;\nabla^{s}\;\rho_{0}\;\theta^{b}(x,t)\;{\dot{\theta }}^{b}{\left(x,t\right)}_{t}\;\delta r\;∆\alpha^{gt}{]}^{T}$$

where Φ represents the attitude error of the sub IMU, δV represents the velocity difference between the main node and the sub-node, $\varepsilon^{s}$ represents the random zero deviation of the sub-node, $\nabla^{s}$ represents the random zero deviation of the sub-node plus measurements, $\rho_{0}$ represents the initial installation error angle of the main node and the sub-node, $\Delta \theta^{b}$(x, t) represents the bending deformation angle of the main node and the sub-node, δr represents the dynamic lever arm of the main node and the sub-node, and $\Delta \alpha^{gt}$ represents the coupling angle between the main node and the sub-node.

The formula of the system state-transition matrix F is shown as follows:

(26) $$\begin{array}{l} F=\left[\begin{array}{ccccccccc} \left(-\omega_{in}^{n}\times \right) & 0_{3\times 3} & -C_{zs}^{n} & 0_{3\times 3} & 0_{3\times 3} & 0_{3\times 3} & 0_{3\times 3} & 0_{3\times 3} & 0_{3\times 3}\\ 0_{3\times 3} & F_{22} & 0_{3\times 3} & C_{zs}^{n} & 0_{3\times 3} & F_{26} & F_{27} & 0_{3\times 3} & F_{29}\\ 0_{3\times 3} & 0_{3\times 3} & 0_{3\times 3} & 0_{3\times 3} & 0_{3\times 3} & 0_{3\times 3} & 0_{3\times 3} & 0_{3\times 3} & 0_{3\times 3}\\ 0_{3\times 3} & 0_{3\times 3} & 0_{3\times 3} & 0_{3\times 3} & 0_{3\times 3} & 0_{3\times 3} & 0_{3\times 3} & 0_{3\times 3} & 0_{3\times 3}\\ 0_{3\times 3} & 0_{3\times 3} & 0_{3\times 3} & 0_{3\times 3} & 0_{3\times 3} & 0_{3\times 3} & 0_{3\times 3} & 0_{3\times 3} & 0_{3\times 3}\\ 0_{3\times 3} & 0_{3\times 3} & 0_{3\times 3} & 0_{3\times 3} & 0_{3\times 3} & 0_{3\times 3} & I_{3\times 3} & 0_{3\times 3} & 0_{3\times 3}\\ 0_{3\times 3} & 0_{3\times 3} & 0_{3\times 3} & 0_{3\times 3} & 0_{3\times 3} & J_{2} & J_{1} & 0_{3\times 3} & 0_{3\times 3}\\ 0_{3\times 3} & 0_{3\times 3} & 0_{3\times 3} & 0_{3\times 3} & 0_{3\times 3} & F_{86} & F_{87} & 0_{3\times 3} & 0_{3\times 3}\\ 0_{3\times 3} & 0_{3\times 3} & C_{zs}^{n} & 0_{3\times 3} & 0_{3\times 3} & F_{96} & F_{97} & 0_{3\times 3} & 0_{3\times 3} \end{array}\right] \end{array}$$

where $F_{22}=-[(2\omega_{ie}^{n}+\omega_{en}^{n})\times ]$, $F_{26}=R_{0}J_{2}+[(\omega_{im}^{n}\times )(\omega_{im}^{n}\times )+({\dot{\omega }}_{im}^{n}\times )]R_{0}$, $F_{86}=R_{0}MJ_{2}$, $F_{27}=(2(\omega_{im}^{n}\times )R_{0}+R_{0}J_{1})$, $F_{29}=[(\omega_{im}^{n}\times )(\omega_{im}^{n}\times )+({\dot{\omega }}_{im}^{n}\times )]R_{0}$, $F_{87}=R_{0}+R_{0}MJ_{1}$, $F_{96}=MJ_{2}$, $F_{97}=MJ_{1}$.

(2) Measurement equation
  (1) Attitude measurements

Attitude measurements are the difference between the attitude angle of the main node and sub-node and are shown as follows.

(27) $$\begin{array}{ll} Y_{a} & =a_{m}-a_{s}\\ & =\delta a+\varpi_{a}\\ & =H_{a}X+\varpi_{a} \end{array}$$

where $\alpha_{m}$ and $\alpha_{s}$ represent the attitude of the main node and the sub-node in navigation coordinates, δa represents the attitude error angle, $\varpi_{\alpha }$ represents the attitude measurement noise, $C_{m}^{n}$ is denoted as $T_{m}$, and ${T_{m}}^{\left(ij\right)}$ represents the elements in the i row and j column of the matrix $C_{m}^{n}$. The attitude measurement matrix $H_{a}$ of the main node and sub-nodes can be expressed as follows:

(28) $$H_{a}=\left[H_{A1}\;0_{3\times 9}\;H_{A2}\;H_{A3}\;0_{3\times 6}\;H_{A4}\right]$$

where $H_{A1}=H_{A4}=\left[\begin{array}{ccc} \frac{{T_{m}}^{\left(12\right)}{T_{m}}^{\left(32\right)}}{{({T_{m}}^{\left(12\right)})}^{2}+({T_{m}}^{\left(22\right)}{)}^{2}} & \frac{{T_{m}}^{\left(22\right)}{T_{m}}^{\left(32\right)}}{{({T_{m}}^{\left(12\right)})}^{2}+({T_{m}}^{\left(22\right)}{)}^{2}} & -1\\ -\frac{{T_{m}}^{\left(22\right)}}{\sqrt{1-({T_{m}}^{\left(32\right)}{)}^{2}}} & \frac{{T_{m}}^{\left(12\right)}}{\sqrt{1-({T_{m}}^{\left(32\right)}{)}^{2}}} & 0\\ \frac{{T_{m}}^{\left(21\right)}{T_{m}}^{\left(33\right)}-{T_{m}}^{\left(31\right)}{T_{m}}^{\left(23\right)}}{{({T_{m}}^{\left(33\right)})}^{2}+({T_{m}}^{\left(31\right)}{)}^{2}} & \frac{{T_{m}}^{\left(31\right)}{T_{m}}^{\left(13\right)}-{T_{m}}^{\left(11\right)}{T_{m}}^{\left(33\right)}}{{({T_{m}}^{\left(33\right)})}^{2}+({T_{m}}^{\left(31\right)}{)}^{2}} & 0 \end{array}\right]$, $H_{A2}=H_{A3}=\left[\begin{array}{ccc} \frac{{T_{m}}^{\left(12\right)}{T_{m}}^{\left(23\right)}-{T_{m}}^{\left(13\right)}{T_{m}}^{\left(22\right)}}{{({T_{m}}^{\left(12\right)})}^{2}+({T_{m}}^{\left(22\right)}{)}^{2}} & 0 & \frac{{T_{m}}^{\left(11\right)}{T_{m}}^{\left(22\right)}-{T_{m}}^{\left(12\right)}{T_{m}}^{\left(21\right)}}{{({T_{m}}^{\left(12\right)})}^{2}+({T_{m}}^{\left(22\right)}{)}^{2}}\\ -\frac{{T_{m}}^{\left(33\right)}}{\sqrt{1-({T_{m}}^{\left(32\right)}{)}^{2}}} & 0 & -\frac{{T_{m}}^{\left(31\right)}}{\sqrt{1-({T_{m}}^{\left(32\right)}{)}^{2}}}\\ -\frac{{T_{m}}^{\left(31\right)}{T_{m}}^{\left(32\right)}}{{({T_{m}}^{\left(33\right)})}^{2}+({T_{m}}^{\left(31\right)}{)}^{2}} & 1 & -\frac{{T_{m}}^{\left(32\right)}{T_{m}}^{\left(33\right)}}{{({T_{m}}^{\left(33\right)})}^{2}+({T_{m}}^{\left(31\right)}{)}^{2}} \end{array}\right]$.

  (2) Velocity measurement

The velocity measurement is the difference in speed between the main node and the sub-node:

(29) $$\begin{array}{ll} Y_{V} & =V_{m}-V_{s}\\ & =\delta V+\varpi_{s}\\ & =H_{s}X+\varpi_{s} \end{array}$$

where $V_{m}$ and $V_{s}$ represent the velocities of the main IMU and the sub IMU under navigation coordinates, respectively, and $\varpi_{s}$ represents the velocity measurement noise. Combined with the coupling error angle model, the velocity measurement matrix $H_{s}$ between the main IMU and the sub IMU can be expressed as follows:

(30) $$H_{s}=\left[0_{3\times 3}\;I_{3\times 3}\;0_{3\times 9}\;H_{V1}\;H_{V2}\;0_{3\times 3}\;0_{3\times 3}\right]$$

where $H_{V1}=R_{0}M(J_{1}J_{2}+J_{2})$, $H_{V2}=R_{0}MJ_{1}^{2}$.

  (3) Angular velocity measurement

Angular velocity measurement is the difference between the angular velocity of the main node and the angular velocity of the sub-nodes, as follows:

(31) $$\begin{array}{ll} Y_{\omega } & =\omega_{m}-\omega_{s}\\ & =\delta \omega +\varpi_{\omega }\\ & =H_{\omega }X+\varpi_{\omega } \end{array}$$

where δω represents the angular velocity error of the main node and sub-nodes, $\varpi_{s}$ represents the angular velocity measurement noise, and the measurement matrix $H_{\omega }$ of the main IMU and sub IMU can be expressed as follows:

(32) $$H_{\omega }=\left[0_{3\times 12}\;\left(\omega_{im}^{m}\times \right)\;\left(\omega_{im}^{m}\times \right)\;I_{3\times 3}\;0_{3\times 3}\;H_{\omega 1}\right]$$

where $H_{\omega 1}=(\omega_{im}^{m}\times +\frac{K(\omega_{\theta })}{\left|\omega_{is}^{s}\right|}\omega_{is}^{s}\times )$.

The measurement equation of the system in the matching method of attitude, velocity, and angular velocity can be expressed as follows:

(33) Y=HX+ϖ

where $Y=\left[\begin{array}{l} Y_{a}\\ Y_{s}\\ Y_{\omega } \end{array}\right]$, $H=\left[\begin{array}{l} H_{a}\\ H_{s}\\ H_{\omega } \end{array}\right]$, $\varpi =\left[\begin{array}{l} \varpi_{a}\\ \varpi_{s}\\ \varpi_{\omega } \end{array}\right]$, ϖ is the measurement noise.

### 2.3. Multi-Sensor Filtering Method

#### 2.3.1. Comparison of Distributed Multi-Sensor Filtering

The measurement of airborne-distributed primary and secondary nodes based on FBG sensors involves the simultaneous operation of one main IMU and multiple sub IMUs. In practical applications, the dynamic and measurement processes in airborne-distributed measurement systems are generally non-linear, and traditional Kalman filtering can no longer meet the accuracy requirements of measurement systems [30]. The research results of distributed multi-node sensor information fusion technology verify its high reliability and fault tolerance. Each sensor is independent and does not affect each other, and multi-node sensor information fusion also has high spatial resolution, which can improve navigation and positioning accuracy. The independent operation of sensors at multiple nodes can increase the dimensionality of the measurement space and reduce observation blind spots. The Kalman filter structures in distributed multi-node sensor information fusion technology mainly include centralized filtering, sequential filtering, distributed filtering, and federated filtering. Centralized filtering transfers all subsystem data to the central processor for analysis, which is computationally complex and has poor fault tolerance, making it difficult to meet real-time requirements. The sequential filtering structure is simple, but it relies on the performance of the first stage and cannot output covariance information. Distributed filtering has the advantages of high reliability, low communication burden, and low energy consumption by locally processing and sharing information among nodes. Moreover, single-node failures do not affect the overall operation of the system, and its robustness is superior to centralized structures.

In practical applications, federated filtering reduces the burden on the central processing unit by dispersing the computation of each child node locally. In addition, the accuracy improvement of the federated filter is reflected in its ability to effectively and dynamically adjust information weights, adaptively adjust based on the estimation accuracy of each node, and optimize the information fusion process. In some scenarios, the accuracy of federated filtering is slightly lower than that of centralized filtering. But in the application of distributed sensing systems, especially in actual high dynamic flight processes, the advantages of federated filtering are worth considering.

#### 2.3.2. Federated Filtering

The federated filtering is a two-stage structure, consisting of a sub-filter that combines the reference system and subsystems. Multiple sub-filters transmit the state estimation and covariance matrix to the main filter, where data fusion is performed and the global optimal estimate is obtained.

(1) Federal filtering formula

The federal filtering formula can be divided into the following four calculation processes:

  (1) Information allocation

(34) $${\hat{X}}_{k-1}^{\left(i\right)}={\hat{X}}_{k-1}^{g}$$

(35) $$P_{k-1}^{\left(i\right)}=\beta_{i}^{-1}P_{k-1}^{g}$$

(36) $$Q_{k-1}^{\left(i\right)}=\beta_{i}^{-1}Q_{k-1}^{g}$$

In the formula, $\beta_{i}$ represents the information allocation coefficient, $0<\beta_{i}<1$, which satisfies the principle of information conservation.

(37) $$\beta_{m}+\sum_{i=1}^{n}\beta_{i}=1$$

  (2) Time update

(38) $${\hat{X}}_{k/k-1}^{\left(i\right)}=\Phi_{k/k-1}^{\left(i\right)}{{\hat{X}}^{\prime }}_{k-1}^{\left(i\right)}$$

(39) $$P_{k/k-1}^{\left(i\right)}=\beta_{i}^{-1}\left[\Phi_{k/k-1}^{\left(i\right)}{P^{\prime }}_{k-1}^{\left(i\right)}{\left(\Phi_{k/k-1}^{\left(i\right)}\right)}^{T}+\Gamma_{k-1}^{\left(i\right)}Q_{k-1}^{\left(i\right)}{\left(\Gamma_{k-1}^{\left(i\right)}\right)}^{T}\right]$$

  (3) Measurement update

(40) $$K_{k}^{\left(i\right)}=P_{k/k-1}^{\left(i\right)}{\left(H_{k}^{\left(i\right)}\right)}^{T}{\left[H_{k}^{\left(i\right)}P_{k/k-1}^{\left(i\right)}{\left(H_{k}^{\left(i\right)}\right)}^{T}+R_{k}^{\left(i\right)}\right]}^{-1}$$

(41) $${\hat{X}}_{k}^{\left(i\right)}={\hat{X}}_{k/k-1}^{\left(i\right)}+K_{k}^{\left(ii\right)}(Z_{k}^{\left(i\right)}-H_{k}^{\left(i\right)}{\hat{X}}_{k/k-1}^{\left(i\right)})$$

(42) $$P_{k}^{\left(i\right)}=(I-K_{k}^{\left(i\right)}H_{k}^{\left(i\right)})P_{k/k-1}^{\left(i\right)}$$

  (4) Information Fusion

Each sub-filter achieves global optimal estimation through information fusion, as follows:

(43) $$P_{k-1}^{g}={\left[\sum_{i=1}^{N}{\left(P_{k-1}^{\left(ci\right)}\right)}^{-1}\right]}^{-1}$$

(44) $${\hat{X}}_{k-1}^{g}=P_{k-1}^{g}\sum_{i=1}^{N}{\left(P_{k-1}^{\left(ci\right)}\right)}^{-1}{\hat{X}}_{k-1}^{\left(ci\right)}$$

(2) Federal filtering mode classification

The different information allocation methods of federated filtering directly affect its structural performance and can be divided into the following modes:

  (1) Zero reset mode

When $\beta_{m}=1$ or $\beta_{n}=0$, it is in zero reset mode. This mode eliminates the time update step, and all information is processed in the main filter, simplifying its computation, but with low fault tolerance.

  (2) Variable proportion mode

When $\beta_{n}=\beta_{m}=1/\left(N+1\right)$, it is in the variable proportion mode. The main filter adopts information in equal distribution. This mode has high accuracy, but subsystem failures can directly affect the operation of other subsystems, reducing fault tolerance.

  (3) No feedback mode

When $\beta_{m}=0$ or $\beta_{n}=1/N$, it is in no feedback mode. The sub-filters in this mode operate independently, while the main filter does not participate in information allocation, resulting in high fault tolerance.

  (4) Fusion feedback mode

The main filter does not participate in information allocation, and the sub-filters receive feedback from the main filter, resulting in an improvement in system accuracy. At this point, when $\beta_{m}=0$ or $\beta_{n}=1/N$, it is in fusion feedback mode. But when the subsystem fails, local filtering is also affected, and the fault tolerance performance will be reduced.

In summary, the fusion feedback mode has higher accuracy and is more suitable for airborne-distributed measurement systems. This article chose the fusion feedback structure as the implementation method of federated filtering.

## 3. Results and Discussion

### 3.1. Federated Adaptive Filtering Method Based on Partition Coefficient

**Figure 9:**
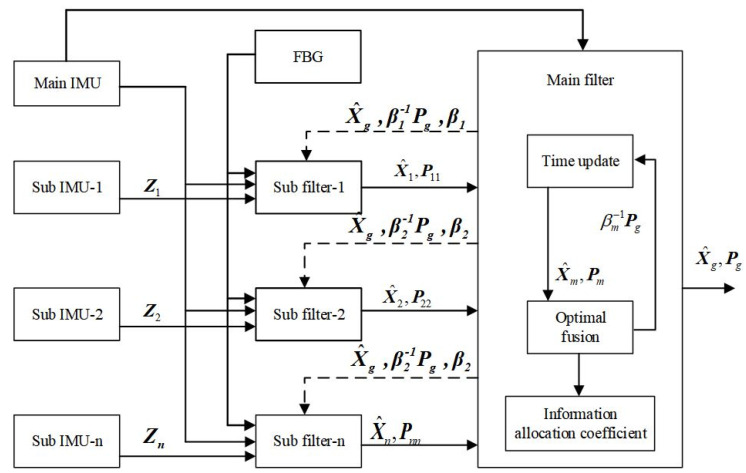
Structural diagram of federated adaptive filtering based on allocation coefficients.

In the airborne-distributed measurement system, the multi-sensor structure composed of the main IMU and sub IMUs faced complex problems, such as non-linear dynamic changes, inconsistent sub-filter accuracy, and frequent environmental disturbances. The traditional distributed federated Kalman filtering method is prone to accumulating system estimation errors, affecting the accuracy and stability of transmission alignment. Therefore, for airborne-distributed transfer alignment, combined with the transfer alignment model based on FBGs and federated filtering, the federated adaptive filtering based on partition coefficients is established, as shown in Figure 9.

In information feedback allocation, the sub-filter allocates weights based on the size of the feedback $\beta_{j}$ from the main filter. In the actual process, the more accurate the estimation of the sub-filter, the greater the weight $\beta_{j}(k)$. Building on this, this paper proposes a federated adaptive filtering method that uses the norm of the error Covariance matrix P to allocate information. The specific methods are as follows. The estimation accuracy of the sub filter j is represented by the norm of P, as shown below.

(45) $$NOP_{j}=\sqrt{tr(P_{j}^{2})}$$

The size of the sub filter $\beta_{j}(k)$ is determined based on the value of ${NOP}_{j}$, the inverse proportional relationship between ${NOP}_{j}$ and $\beta_{j}\left(k\right)$ is defined, and the expression of $\beta_{j}(k)$ is shown as follows:

(46) $$\beta_{1}(k)=\frac{NOP_{2}+NOP_{3}}{2\sum_{j=1}^{3}NOP_{j}}$$

(47) $$\beta_{2}(k)=\frac{NOP_{1}+NOP_{3}}{2\sum_{j=1}^{3}NOP_{j}}$$

(48) $$\beta_{3}(k)=\frac{NOP_{2}+NOP_{1}}{2\sum_{j=1}^{3}NOP_{j}}$$

The effectiveness of the federated adaptive filtering method based on partition coefficients in the distributed transfer alignment of wings is verified through MATLAB 2017a simulation experiments. Firstly, the carrier flight trajectory is obtained from the main IMU data during actual flight. The initial speed of the carrier in all three directions is 0.5 m/s, the initial attitude is ${\left[144.7{}^{\circ}-0.369{}^{\circ}-1.52{}^{\circ}\right]}^{T}$, and the initial position is ${\left[109.25{}^{\circ}\;39.17{}^{\circ}-394.1m\right]}^{T}$.

**Figure 10:**
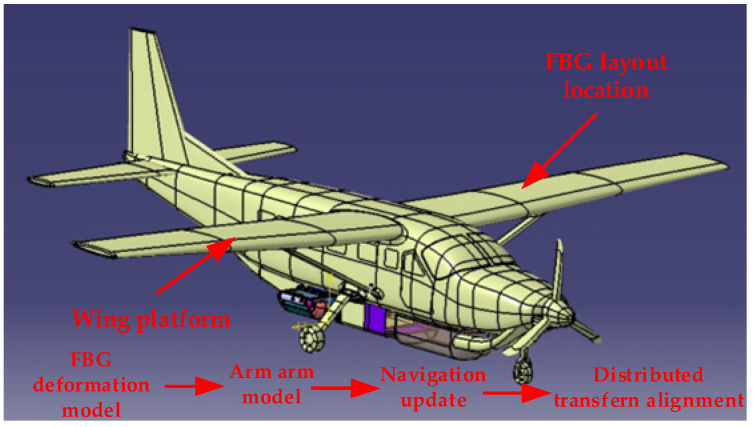
Flight experiment plan.

**Figure 11:**
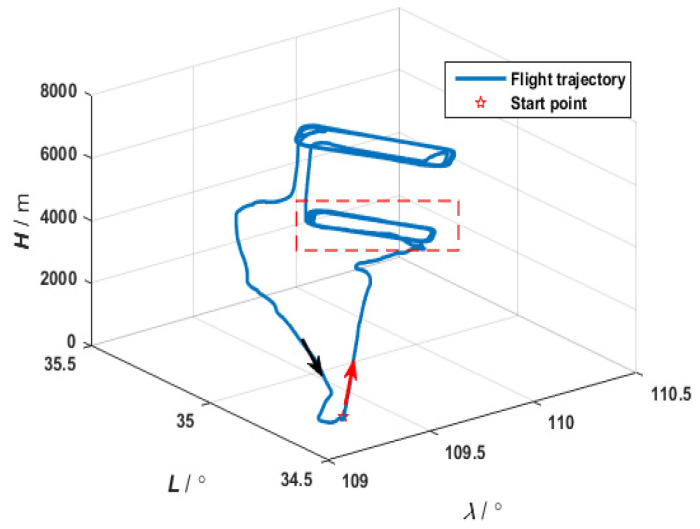
Flight trajectory.

Figure 10 and Figure 11, respectively, show the flight experiment scheme and the flight trajectory designed in this paper. According to the layout scheme in the figure, data from the main IMU and sub IMU were collected during the flight to simulate actual working conditions. The set sub-system parameters simulated the neutron IMU data during flight. The constant drift of the gyroscope in all three directions was $0.05^{{}^{\circ}}/\sqrt{h}$. The random walk of the gyroscope in all three directions was $0.025^{{}^{\circ}}/\sqrt{h}$. The constant zero deviation of the accelerometer in all three directions was 150 μg. The velocity random walk in all three directions of the accelerometer was 50 $\mu g/\sqrt{Hz}$. The sampling frequency was 100 Hz. The initial attitude error was ${\left[30^{\prime }\;30^{\prime }\;3\right]}^{T}$. The initial velocity error was 0.1 m/s. The initial installation angle error was ${\left[0.5^{\prime }\;0.5^{\prime }\;0.5^{\prime }\right]}^{T}$. The coupling angle error was ${\left[10^{\prime }\;10^{\prime }\;10^{\prime }\right]}^{T}$. The error of the lever arm was ${\left[{0.05\;m}^{\prime }\;{0.05\;m}^{\prime }\;{0.05\;m}^{\prime }\right]}^{T}$. The initial lever arms of the three sub-nodes were set as ${\left[{0.85\;m}^{\prime }\;{0.15\;m}^{\prime }\;{0.25\;m}^{\prime }\right]}^{T},\;{\left[{2\;m}^{\prime }\;{0.15\;m}^{\prime }\;{0.28\;m}^{\prime }\right]}^{T}$, and ${\left[{2.65\;m}^{\prime }\;{0.15\;m}^{\prime }\;{0.31\;m}^{\prime }\right]}^{T}$.

**Figure 12:**
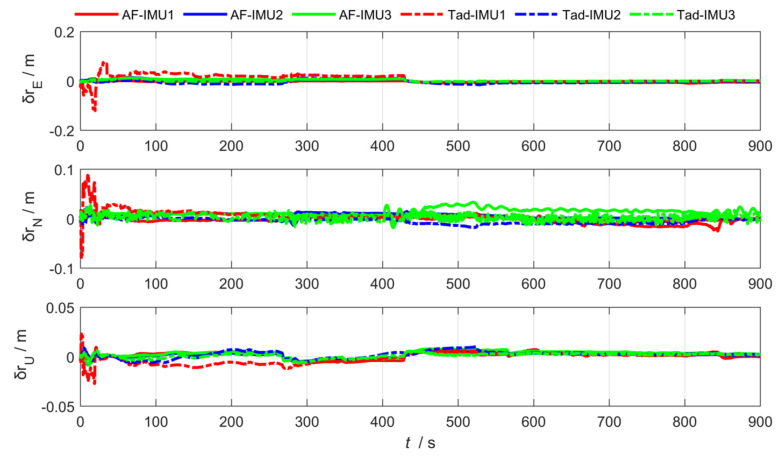
Arm estimation error via federated adaptive filtering based on allocation coefficients.

**Figure 13:**
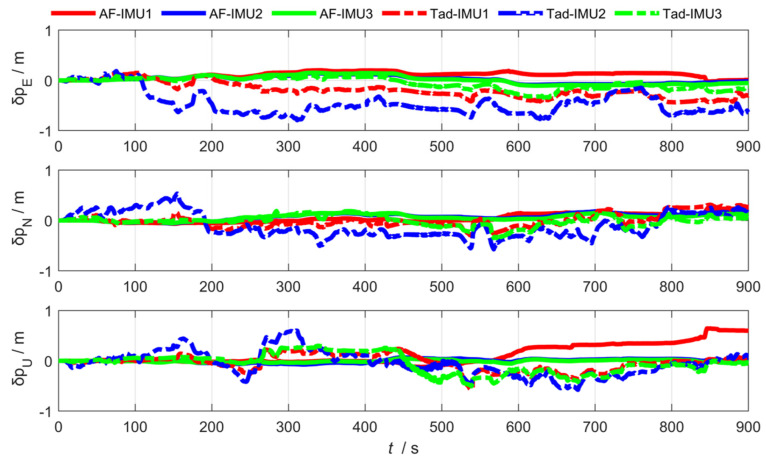
Position estimation error via federated adaptive filtering based on allocation coefficients.

**Figure 14:**
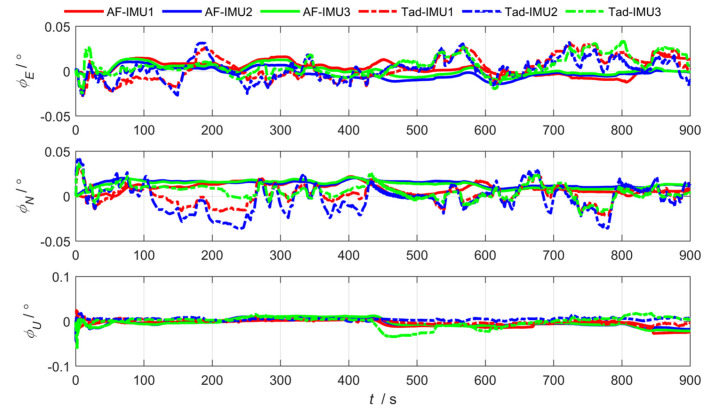
Attitude estimation error via federated adaptive filtering based on allocation coefficients.

As shown in Figure 12, Figure 13 and Figure 14, the abbreviation AF represents the federated adaptive Kalman filter based on allocation coefficients, and the abbreviation Tad represents the traditional Kalman filter. The simulation results of the federated adaptive filtering method based on partition coefficient and the traditional method are shown as follows:

To analyze the effectiveness of the proposed method, the root-mean-square deviation of attitude, position, and lever arm is summarized as follows. Where East (east direction), North (north direction), and Up (sky direction), respectively, represent the three-dimensional spatial coordinate axis directions where the position error and lever arm error are located; and Pitch, Roll, and Yaw are attitude angle parameters corresponding to the three directions.

As shown in Table 1 and Table 2, the experimental results show that the federated adaptive algorithm based on partition coefficients can improve the accuracy of motion parameters compared with traditional algorithms. Selecting RMSE as the evaluation standard, the pitch angle accuracy of sub IMU attitude estimation improved by 66.38%. The accuracy of the heading and roll angle slightly decreased. It can be concluded that the position estimation accuracy improved by 75.67%. The accuracy of arm estimation is significantly better than traditional algorithms in the east and up directions, while the accuracy in the north direction decreased.

In conclusion, for the distributed federated Kalman filter with adaptive information partition coefficients, the online adjustment of information partition coefficients promoted the anti-interference performance of the overall filter. In the actual process, the estimation of the sub-filter that is least affected by the outside world is more accurate and the partition coefficient $\beta_{j}(k)$ of the information should be larger, so the probability of the main filter being polluted by the wrong information pollution when performing the optimal information fusion is smaller, and the ability to correct the lever arm, position, and attitude is stronger.

### 3.2. Federated Adaptive Filtering Method Based on the R Update

**Figure 15:**
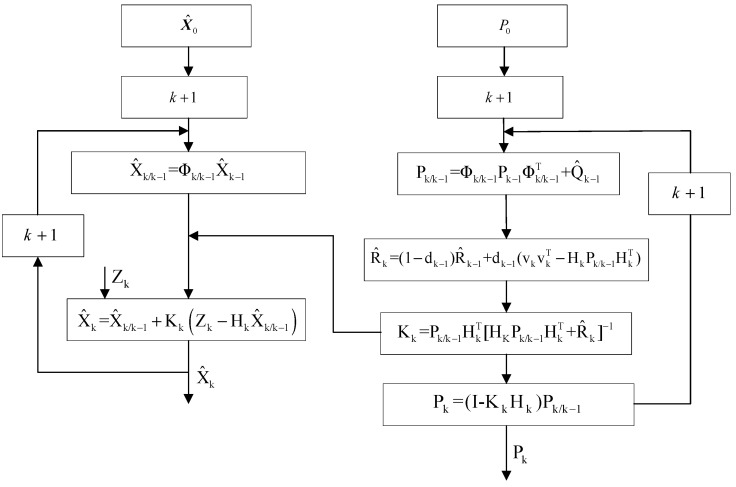
Flow diagram of a federated sub-filter based on the R update.

In practical application, the sub-filter of the federated filtering method has some errors when using the traditional Kalman filter filtering method to obtain information, and the uncertainty of the system noise variance matrix **Q** and the observation noise variance matrix **R** can lead to the reduction of the filtering accuracy. The measurement noise in airborne-distributed transfer alignment has uncertainty. In order to reduce the computational complexity of the filtering algorithm and increase its stability, a federated adaptive filtering method that only estimates is proposed. Specific improvements are shown in Figure 15.

**Figure 16:**
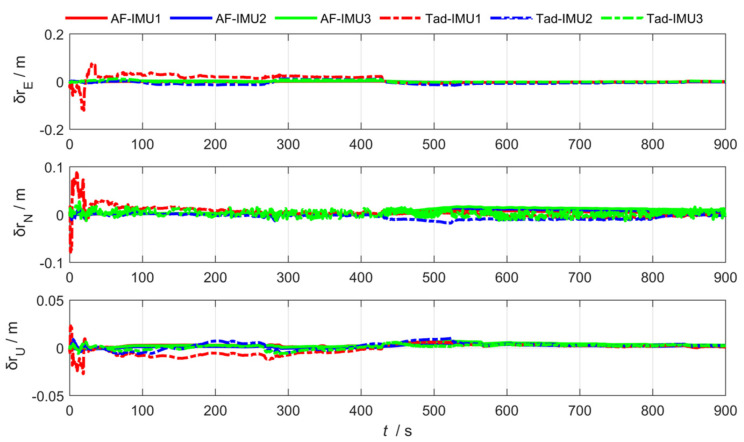
Arm estimation error via federated adaptive filtering based on the R update.

**Figure 17:**
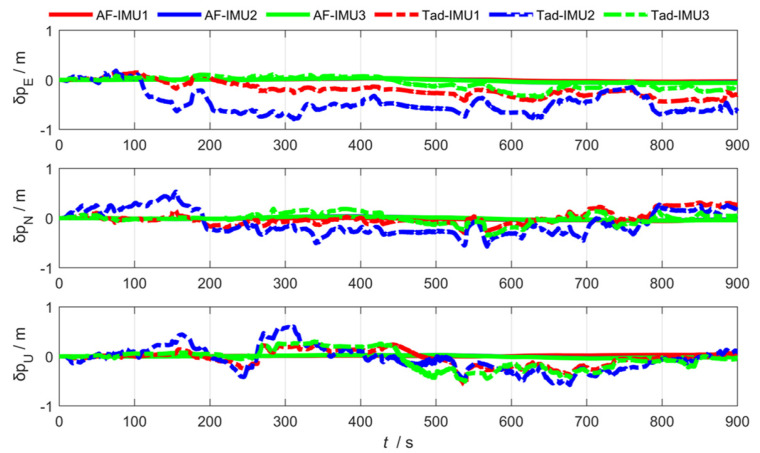
Position estimation error via federated adaptive filtering based on the R update.

**Figure 18:**
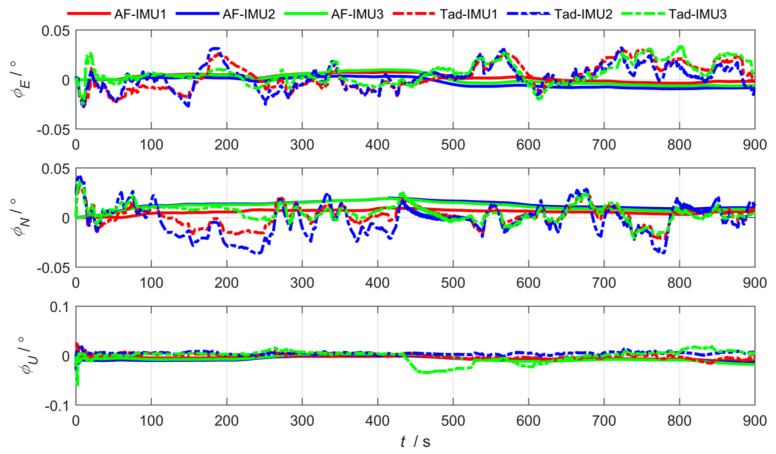
Attitude estimation error via federated adaptive filtering based on the R update.

The formula for the federated sub filter algorithm based on **R** update is summarized as follows. The basic filtering part is as follows:

(49) $${\hat{X}}_{k/k-1}=\Phi_{k/k-1}{\hat{X}}_{k-1}+{\hat{q}}_{k}$$

(50) $$v_{k}=Z_{k}-H_{k}{\hat{X}}_{k/k-1}-{\hat{r}}_{k}$$

(51) $${\hat{X}}_{k}={\hat{X}}_{k/k-1}+K_{k}v_{k}$$

(52) $$P_{k/k-1}=\Phi_{k/k-1}P_{k-1}\Phi_{k/k-1}^{T}+\Gamma_{k-1}{\hat{Q}}_{k-1}\Gamma_{k-1}^{T}$$

(53) $$K_{k}=P_{k/k-1}H_{k}^{T}{\left[H_{K}P_{k/k-1}H_{k}^{T}+{\hat{R}}_{k}\right]}^{-1}$$

(54) $$P_{k}=(I-K_{k}H_{k})P_{k/k-1}$$

where $q_{k}$ represents the system noise mean of a linear discrete system, $r_{k}$ represents the measurement noise mean, $Q_{k}$ represents the system noise variance matrix, and $R_{k}$ represents the measurement noise variance matrix. The **R** update section is as follows:

(55) $${\hat{R}}_{k}=(1-d_{k-1}){\hat{R}}_{k-1}+d_{k-1}(v_{k}v_{k}^{T}-H_{k}P_{k/k-1}H_{k}^{T})$$

(56) $$d_{k}=\left(1-b\right)/\left(1-b^{k}\right)$$

where $1-d_{k-1}$ and, $d_{k-1}$ represent the adaptive coefficient. The smaller the value of $d_{k-1}$, the less dependence on outdated noise, and b is the forgetting factor, with a value of 0.95≤b≤0.99. To verify the effectiveness of the **R** update-based federated adaptive filtering algorithm, the experimental results compared with the traditional algorithm are shown in Figure 16, Figure 17 and Figure 18.

To analyze the effectiveness of the federated adaptive filtering algorithm based on the **R** update, the attitude, position, and lever arm root-mean-square deviation are summarized as follows:

As shown in Table 3 and Table 4, Compared with the traditional algorithm, the federated adaptive algorithm based on the **R** update improved the pitch angle accuracy of sub IMUs by 76.72%, while the heading and roll angle accuracy slightly decreased. The position estimation accuracy and lever arm estimation accuracy improved in all three directions, by 63.51% and 1.52%, respectively.

The federated adaptive filtering method based on the **R** update separates effective information from time-varying noise in measurement information, accurately estimates the noise variance matrix $R_{k}$, and adjusts the filter gain $Z_{k}$ in real time, ultimately improving system accuracy. Compared to traditional methods, the estimation accuracy of position, attitude, and lever arm has improved. However, the overall system has weak anti-interference ability. After the sub-filter is affected, the main filter is affected by similar disturbances during information fusion, which reduces the accuracy of sub IMU motion parameter estimation. Compared to traditional methods, the estimation accuracy of position, attitude, and lever arm has improved.

## 4. Conclusions

In this paper, the transfer alignment model was established, and a federated adaptive filtering algorithm was proposed by combining federated filtering with FBG-assisted transfer alignment. The first method is a federated adaptive filtering method that uses the norm of the error Covariance matrix P to allocate information. The experimental results show that, compared to traditional algorithms, the pitch angle accuracy and position estimation accuracy of the sub IMU improved by 66.38% and 75.67%, respectively. The arm estimation accuracy is significantly better in the east and up directions. The second method is a federated adaptive filtering method based on the R update, which addresses the impact of time-varying characteristics of measurement noise on the filter. Experimental results show that, compared to traditional algorithms, the IMU pitch angle accuracy, position estimation accuracy, and lever arm estimation accuracy improved by 76.72%, 63.51%, and 1.52%, respectively.

## Figures and Tables

**Table 1:** Position and lever arm estimation accuracy via federated adaptive filtering based on allocation coefficients.

IMU	Parameter	Method	RMSE		
			East	North	Up
IMU 1	Position error (m)	Traditional Kalman Filter	0.267	0.148	0.157
		Federated adaptive filtering based on partition coefficients	0.013	0.023	0.016
	Arm error (m)	Traditional Kalman Filter	0.018	0.012	0.005
		Federated adaptive filtering based on partition coefficients	0.002	0.006	0.001
IMU 2	Position error (m)	Traditional Kalman Filter	0.543	0.262	0.246
		Federated adaptive filtering based on partition coefficients	0.028	0.035	0.015
	Arm error (m)	Traditional Kalman Filter	0.008	0.007	0.004
		Federated adaptive filtering based on partition coefficients	0.003	0.011	0.002
IMU 3	Position error (m)	Traditional Kalman Filter	0.151	0.100	0.198
		Federated adaptive filtering based on partition coefficients	0.022	0.024	0.015
	Arm error (m)	Traditional Kalman Filter	0.004	0.007	0.003
		Federated adaptive filtering based on partition coefficients	0.003	0.012	0.002

**Table 2:** Attitude estimation accuracy via federated adaptive filtering based on allocation coefficients.

IMU	Parameter	Method	RMSE		
			Pitch	Roll	Yaw
IMU 1	Attitude error (°)	Traditional Kalman Filter	0.0131	0.0010	0.0050
		Federated adaptive filtering based on partition coefficients	0.0028	0.0061	0.0063
IMU 2	Attitude error (°)	Traditional Kalman Filter	0.0116	0.0150	0.0053
		Federated adaptive filtering based on partition coefficients	0.0039	0.0096	0.0077
IMU 3	Attitude error (°)	Traditional Kalman Filter	0.0125	0.0094	0.0116
		Federated adaptive filtering based on partition coefficients	0.0032	0.0101	0.0080

**Table 3:** Position and lever arm estimation accuracy via federated adaptive filtering based on the R update.

IMU	Parameter	Method	RMSE		
			East	North	Up
IMU 1	Position error (m)	Traditional Kalman Filter	0.267	0.148	0.157
		Federated adaptive filtering based on the R update	0.012	0.027	0.014
	Arm error (m)	Traditional Kalman Filter	0.018	0.012	0.005
		Federated adaptive filtering based on the R update	0.001	0.002	0.001
IMU 2	Position error (m)	Traditional Kalman Filter	0.543	0.262	0.246
		Federated adaptive filtering based on the R update	0.031	0.045	0.017
	Arm error (m)	Traditional Kalman Filter	0.008	0.007	0.004
		Federated adaptive filtering based on the R update	0.003	0.006	0.001
IMU 3	Position error (m)	Traditional Kalman Filter	0.151	0.100	0.198
		Federated adaptive filtering based on the R update	0.025	0.037	0.015
	Arm error (m)	Traditional Kalman Filter	0.004	0.007	0.003
		Federated adaptive filtering based on the R update	0.002	0.007	0.001

**Table 4:** Attitude estimation accuracy via federated adaptive filtering based on the R update.

IMU	Parameter	Method	RMSE		
			Pitch	Roll	Yaw
IMU 1	Attitude error (°)	Traditional Kalman Filter	0.0131	0.0010	0.0050
		Federated adaptive filtering based on the R update	0.0025	0.0057	0.0063
IMU 2	Attitude error (°)	Traditional Kalman Filter	0.0116	0.0150	0.0053
		Federated adaptive filtering based on the R update	0.0027	0.0087	0.0075
IMU 3	Attitude error (°)	Traditional Kalman Filter	0.0125	0.0094	0.0116
		Federated adaptive filtering based on the R update	0.0023	0.0091	0.0076

## Data Availability

The data presented in this study are available on request from the corresponding author.
